# A Review of the Characteristics, Synthesis, and Thermodynamics of Type-II Weyl Semimetal WTe_2_

**DOI:** 10.3390/ma11071185

**Published:** 2018-07-10

**Authors:** Wenchao Tian, Wenbo Yu, Xiaohan Liu, Yongkun Wang, Jing Shi

**Affiliations:** School of Electro-Mechanical Engineering, Xidian University, Number 2 Taibai South Road, Xi’an 710071, China; wctian@xidian.edu.cn (W.T.); liuxiaohan_xidian@foxmail.com (X.L.); ykwang@xidian.edu.cn (Y.W.); jshi@xidian.edu.cn (J.S.)

**Keywords:** Weyl semimetal, WTe_2_, characteristics, synthesis, thermodynamics

## Abstract

WTe_2_ as a candidate of transition metal dichalcogenides (TMDs) exhibits many excellent properties, such as non-saturable large magnetoresistance (MR). Firstly, the crystal structure and characteristics of WTe_2_ are introduced, followed by a summary of the synthesis methods. Its thermodynamic properties are highlighted due to the insufficient research. Finally, a comprehensive analysis and discussion are introduced to interpret the advantages, challenges, and future prospects. Some results are shown as follows. (1) The chiral anomaly, pressure-induced conductivity, and non-saturable large MR are all unique properties of WTe_2_ that attract wide attention, but it is also a promising thermoelectric material that holds anisotropic ultra-low thermal conductivity (0.46 W·m^−1^·K^−1^). WTe_2_ is expected to have the lowest thermal conductivity, owing to the heavy atom mass and low Debye temperature. (2) The synthesis methods influence the properties significantly. Although large-scale few-layer WTe_2_ in high quality can be obtained by many methods, the preparation has not yet been industrialized, which limits its applications. (3) The thermodynamic properties of WTe_2_ are influenced by temperature, scale, and lattice orientations. However, the in-plane anisotropy cannot be observed in the experiment, as the intrinsic property is suppressed by defects and boundary scattering. Overall, this work provides an opportunity to develop the applications of WTe_2_.

## 1. Introduction

Weyl semimetal (WSM) is a new state of matter in condensed matter physics that contains Weyl fermions acting as emergent quasiparticles. Its bulk material owns the Fermi arc surface state at the boundary, and this novel electronic structure makes it hold characteristics of topological properties. In 2016, many researchers reported that the Fermi arcs on its surface, and that Fermi cones and Weyl nodes in the bulk of WSM could be observed through photoelectron spectroscopy directly [1,2,3,4]. Also, WSM is a new generation of topological insulators with unique band structures on the surface and in the bulk, which means that it is different from the topological insulators that are characteristic of gapless surface states protected by time-reversal symmetry. In addition, the robust topological states at the step edge of WTe_2_ can be directly observed by spectroscopic-imaging scanning tunneling microscopy (STM) in the experiment, which validates the prediction by first principle theory [5]. The WSM phase stands between the general phase and the topological phase, and the effective band is a low-energy linear Dirac-type dispersion. The Weyl fermions in the bulk can be utilized to study the chiral anomaly in the condensed matter physics field; meanwhile, it owns huge unsaturated magnetoresistance (MR), which makes it a promising type of material. Since the discovery of non-magnetic WSM including TaAs, TaP, NbAs, and NbP by Weng et al. in 2015, it has found that they are unique from the traditional magnetic WSM in terms of electromagnetism [6]. The type-I WSM, such as TaAs, produces a large non-saturated negative MR in parallel electric and magnetic fields, whereas type-II WSM such as WTe_2_ possesses a positive MR that is related to crystal orientations. It should be noted that spatial inversion symmetry cannot be observed in TaAs, and the position of the k-space of the Fermi arc terminal on the surface matches the projection of numerous Weyl nodes on the surface Brillouin zone. Furthermore, the Fermi arc on the surface of WSM contributes to the quantum oscillations of magnetic transport. As a result, its excellent electromagnetic property also attracts many researchers, but meanwhile, WSM is also a promising thermoelectric material. Specifically, it holds a high thermoelectric figure of merit and low thermal conductivity, so it is potentially used in thermoelectric fields. However, there are few studies in this field.

WTe_2_ as a non-magnetic thermoelectric semimetal is a recently discovered type-II WSM. It has become a research hotspot owing to its huge non-saturated MR and chiral anomaly. Except for graphene, the limitations of other two-dimensional (2D) atomic-level thickness metals such as β-MoTe_2_ and WTe_2_ are studied insufficiently. They are stable as semimetals in the deformation of the octahedral 1T (CdI_2_) geometry, possessing an in-plane bending chain formed by Mo/W atom pairs dimerized in charge exchange between metals, and Van der Waals forces play a role in the bonding interactions among dominant layers [7]. MoTe_2_ can be synthesized in 2H and 1T polytypes or reversibly converted between the two polytypes as a function of temperature or strain. Since the 1960s, WTe_2_ has been proven that it has an orthogonal structure with a space group Pmn21 (Td), and it is one of the most promising dichalcogenides in the VI groups. The 2D transition metal dichalcogenides (TMD) materials (AB_2_: A is W or Mo; B is S, Se or Te) can be used in applications of low-dissipation electronics, spintronics, optoelectronics, and catalysis. Recently, it is also well known due to many excellent properties including unsaturated MR along the a-axis, superconductivity induced by pressure, spin-orbit structure, temperature-driven Lifshitz transition, and low-energy light absorption [8]. The spin-orbit out-of-plane field-like torque is related to the thickness significantly, but the magnitude of the out-of-plane antidamping torque is weakly influenced by thickness [9]. In addition, a novel Weyl fermion is observed in WTe_2_ firstly that is different from conventional one, and the monolayer material has a quantum spin Hall effect. However, in addition to electro-optical properties, the thermal properties are also critical, because it determines the heat dissipation efficiency, which is a significant parameter for the performance and reliability of devices.

The WSM is a new generation of topological semimetal materials developed from topological insulators, and its research is still in its infancy. Many of its researches are based on its electromagnetic properties, and there are few studies on its thermodynamic properties. The WTe_2_ is one of the most valuable materials in the thermodynamic field, so this paper summarizes its main features, and the researches of thermodynamics are mainly summarized and analyzed as a supplement of studies, which is helpful for the development of this new material.

## 2. Structure and Characteristics of WTe_2_

### 2.1. Crystal Structure and Surface Structure

**Figure 1:**
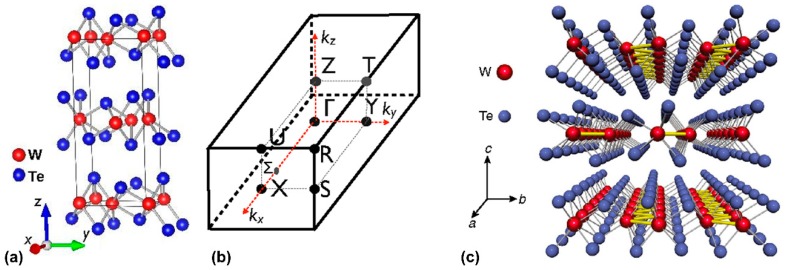
(**a**) The crystal structure of WTe_2_ along the x-axis, y-axis, and z-axis; (**b**) The Brillouin zone of the rhombohedral unit cell [12]; (**c**) The crystal structure of WTe_2_ in ab-plane and along c-axis [10].

WTe_2_ is a typical candidate of layered TMDs [10]. Its distorted 1T (Td) structure can be observed by atomic resolution imaging in the bulk material at 300 K, which is different from most TMDs (2H or 1T) [11]. Figure 1 shows the layered crystal structure of WTe_2_ and its Brillouin zone [12]. Its crystal structure can be regarded as the distortion of MoS_2_ along the a-axis (W chain). It is similar to graphite to some extent in that it is composed of many layers, and the W layers are separated from each other by two layers of Te, which is stacked along the z-axis. The number of layers can be observed by the Raman modes in Raman spectroscopy [13]. The lattice structure is orthorhombic, and the space group is Pmn21 (C^7^_2v_). The distance between adjacent W atoms is smaller along the x-axis than that along the y-axis or the z-axis, which results in strong anisotropy. The topological surface state exists because all of the Weyl points (WPs) are projected onto different points for the (001) surface.

WTe_2_ and β-MoTe_2_ are typical semimetals because the displacement of W and Mo atoms from the octahedron center of Te atoms compensates for the loss of band structure. Actually, the crystal structure of Td-WTe_2_ is similar to that of β-MoTe_2_. The coordination of a distorted octahedron of Te atoms around the W atoms displaces them from the octahedron center in the ab-plane, but the layered configuration still exists along the c-axis, as shown in Figure 1c. This distorted structure contributes to a rise of the coordination number (from six to eight) for the tungsten. However, their stacking layers are different from that of an orthorhombic symmetry and a monoclinic symmetry, which are shown in WTe_2_ (Pmn21) and β-MoTe_2_ (P21/m), respectively [14]. Furthermore, 2H and 1T are common crystal structures for TMDs such as MoTe_2_. A trigonal prismatic coordination (six chalcogen atoms) is around the Mo atoms, and a slight elongation of the prism is shown vertically to the layers, which represents the 2H-polytype of MoTe_2_. In addition, the 2H structure consists of three hexagonal atoms planes (chalcogen-metal-chalcogen) with a van der Waals force between layers along the c-axis, and strong covalent bonds connect the in-plane atoms. The Madelung energy of Td-WTe_2_ is lower than that of assumed 2H-WTe_2_, which is the reason for the semimetal state [15]. Another typical structure of TMDs is 1T octahedral coordination, which is different from 2H in that the chalcogen atoms are located directly above each other in the trigonal prism. The W atoms are octahedrally coordinated by Te atoms for the 1T structure, but the sandwich layers are inverse for distorted 1T (Td). The displacement of the W atoms (horizontally by 0.95 Å toward the top Te chain and perpendicularly by 0.21 Å vis a vis the bottom W atom) from the octahedral center is related to the strong intermetallic bonding so that pairs can form, and a zig-zag pattern is also shown in crystal [16].

It is widely known that dichalcogenides such as WTe_2_ are suitable for the applications in environments with extreme temperatures and pressures in engineering, and they also can be employed in semiconductors, batteries, catalysts, etc. The dichalcogenides typically perform a layered structure of the compound in which a metal atomic layer is sandwiched between two chalcogen atoms, as shown in Figure 1a. Also, the chemical bonds of the atoms are relatively stable in the in-plane layer, while the interaction between out-of-plane layers is weak, owing to the connection of the van der Waals force. Therefore, the cracking tends to occur in this structure, and considerable anisotropy is shown in many physical properties. In addition, the distance between layers is wide enough to be doped by impurities, so the WTe_2_ is easy to be influenced by doping. A twisted octahedral coordination around tungsten atoms is performed in the structure that is in line with the morphology of the form of MoTe_2_ under high temperature. Its structure is different from most TMDs, which are characterized by the triangular prism or monoclinic structure. Furthermore, it is obvious from Figure 1a that the layers are curved, resulting from the dislocation of W atoms from the center of the octahedron, which forms a chain extending through the crystal. It is worth mentioning that the displacement of the W atom enhances the strength of metallic bond so that the conduction of WTe_2_ is improved, and it also contributes to a novel electronic band structure, which is the reason why WTe_2_ has a relatively high conductivity in dichalcogenides. It should be noted that some properties of the type-I and type-II WSMs are very different, such as the thermodynamic properties.

**Figure 2:**
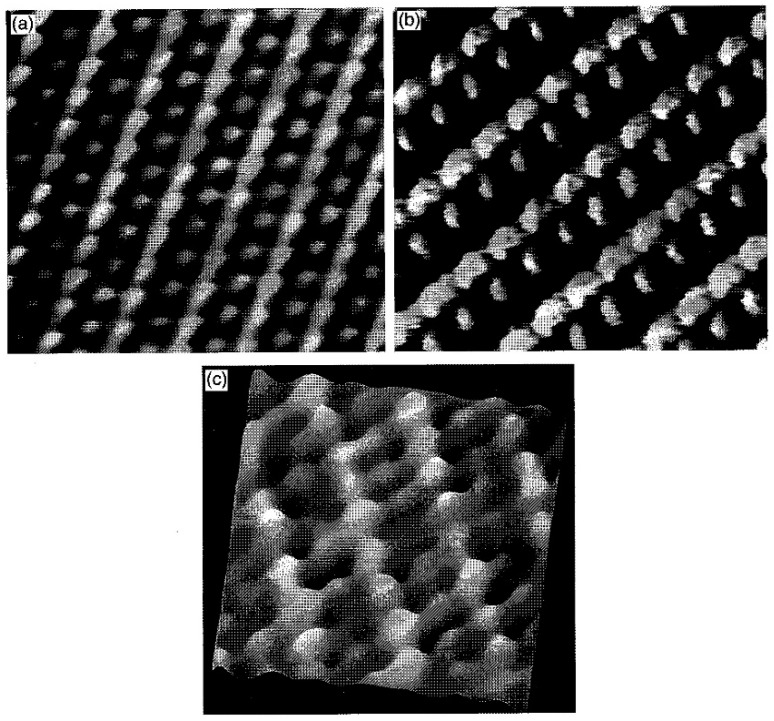
(**a**) Te-layer-like scanning tunneling microscopy (STM) image (37 × 32 Å^2^) of WTe_2_ (001) surface; (**b**) W-layer-like STM image (30 × 25 Å^2^) of WTe_2_ (001) surface; (**c**) Corresponding atomic force microscopy (AFM) image (21 × 21 Å^2^, lateral force mode) [17], with copyright permission from © Elsevier.

The surface structure of WTe_2_ is also important, and it is significantly associated with the unique features, such as the emergence of MR. The tellurium and tungsten layers are buckled in the direction of the c-axis, but the tellurium and tungsten atoms do not sit in the identical plane in every layer. In 1994, Crossley et al. studied the surface of WTe_2_ with [001] orientation in ultra-high vacuum by scanning tunneling spectroscopy (STS), and found that it depended on the polarity of tunnel voltage [16]. The tip effect might cause artefacts, which explained the STM images. In 1996, Hla et al. investigated the positions of atoms on the (001) surface of TMDs by varying the metal–Te distances [17]. The STM images in Figure 2a,b show the detailed corrugated Te atoms on the top and metal layers, and the results were compared with those by atomic force microscopy (AFM) in Figure 2c. They stated that only the Te-layer-like images were observed when the tip was far from the surface, and the results were not associated with the polarity of bias voltage, which was not in line with the theoretical results (observation was available only at the negative bias). In 2017, the atomic structure of the WTe_2_ surface was studied by Kawahara et al. by means of low-energy electron diffraction (LEED), STM, and density functional theory (DFT) [18]. In the first layer, a tiny surface relaxation was found, and it decayed in an oscillatory mode. In addition, they suggested that the topology of the Fermi surface was affected by the relaxation.

### 2.2. Classification

WSM is mainly divided into two types in accordance with fermions: type-I (TaAs, TaP, NbAs, and NbP) and type-II (WTe_2_ and MoTe_2_). Standard WPs exists on the spotted Fermi surface of Type-I WSM. By contrast, the type-II WPs are still the protected intersections, but occur at the boundary between the electron and hole pockets [12]. This is the reason why many properties are different for these two types of WSMs, and type-II WSM holds some novel physical phenomena as well, such as the open Fermi arcs existing on the Fermi surface, and planar orientation-dependent chiral anomaly [10,12].

### 2.3. Novel Weyl Fermions, Fermi Arcs, and Surface State

**Figure 3:**
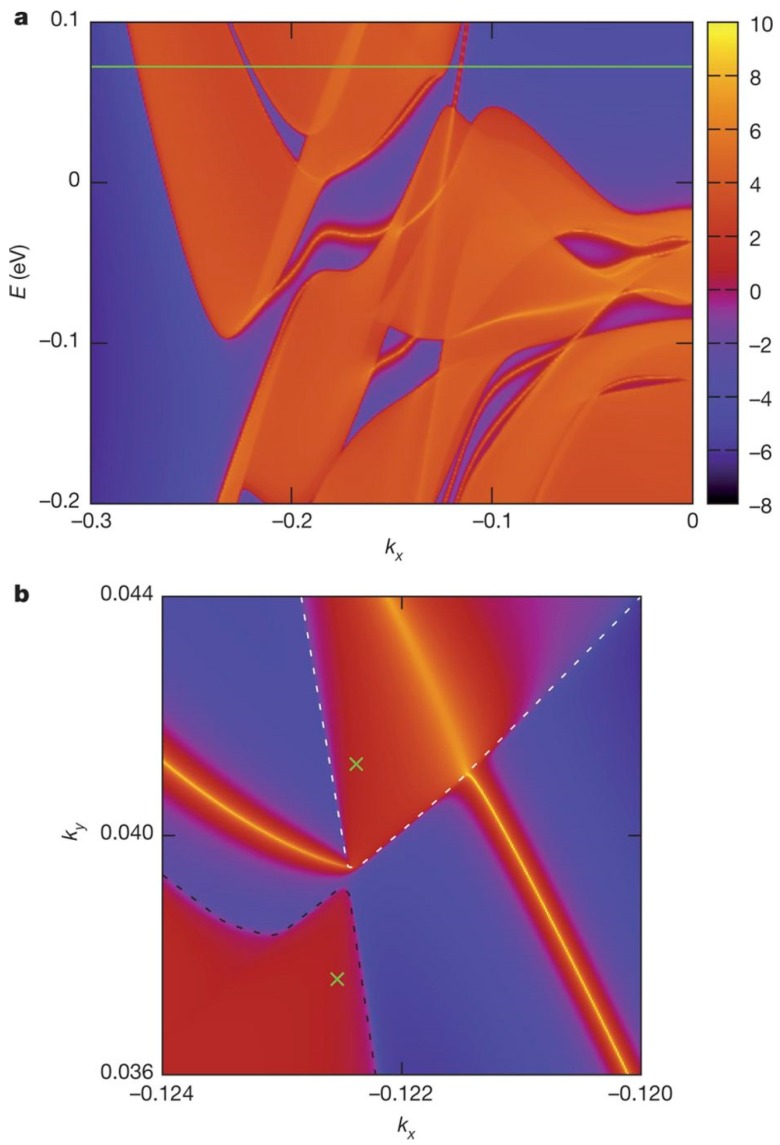
(**a**) The position of the Fermi level (green line); (**b**) The position of Weyl points (WPs) and Fermi arc on the Fermi surface [12].

The fermion is a fundamental component in the atom, and is divided into three types as basic particles: Dirac, Majorana, and Weyl [12]. However, Majorana and Weyl fermions can only be observed in the state of low-energy excitation, so they are rarely observed in the experiments, but the discovery of topological superconductors and semimetals allows the observation of them in the experiments. Weyl fermions in the boundaries of hole pockets and electrons have been ignored because of the contravention of Lorentz symmetry in high-energy physics. By contrast, this theory is not included in condensed matter physics, and new Weyl fermions can be found by promoting Dirac equations. In monolayer WTe_2_, the special electron–hole-pocket structure cannot be observed due to the absence of interlayer interactions [19]. However, it can be modified by the transition-metal Ni-adsorbed system in accordance with density functional theory. Figure 3 shows the positions of the Fermi level and WP on the (001) surface [12]. The green line is the location of the Fermi level that is between the WPs, as shown in Figure 3a. In addition, the two crosses represent the WPs in Figure 3b, and it also shows a Fermi arc that connects the hole and electron pocket on the Fermi surface.

**Figure 4:**
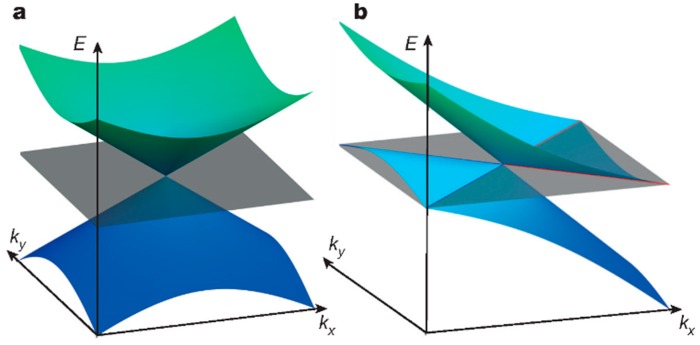
The WP and the Fermi surface of type-I (**a**) and type-II WSM (**b**) [12].

WTe_2_ owns a complex band structure and unique hole Fermi pockets, and this special electronic structure contributes to many novel characteristics, such as large MR. In Figure 4, two bands (green and blue) of the WSM cross at the Fermi level linearly, while the number of bands doubles for Dirac semimetal (DSM) [12]. Figure 4a illustrates the WP and punctate Fermi level of type-I WSM, while the WP exists at the boundaries between electron and hole pockets (blue/red lines) for type-II WSM, as shown in Figure 4b. The Fermi level is marked as grey flat, clearly. Also, the Weyl or the gapless Dirac equation can be used to describe respective Hamiltonian intersections. An interesting thing is that WPs generally appear in pairs, and the reason for this is that the net charge cannot exist throughout the Brillouin zone [20]. In addition, they cannot be affected by slight perturbations, and the electron pocket shows opposite behavior [21]. There are totally four pairs of WPs that are located between the Fermi levels of 0.052 eV and 0.058 eV. Furthermore, the Fermi arc is on the Fermi surface, and the cone-shaped spectrum is tilted due to dynamics, which does not follow the Lorentz invariance of the Weyl fermions in the quantum field. This special case was neglected before the discovery of WSM, but it proved the correctness of the existence of type-II WSM. Moreover, the tilted spectrum is associated with the dynamics in the spectrum as mentioned, specifically, the dynamics dominate in the exact direction in reciprocal space (potential component accounts for a small part). Then, the tilt is large enough for WP to appear at the boundary of electron and hole pockets, which is inverse to the case of type-I WSM (the particular direction cannot be found). Another difference is that type-II WSM does not show chiral anomaly under magnetic field in any directions, and this phenomenon can be observed only in which the magnetic field direction is in the cone.

In recent years, the electronic structures of WTe_2_, including WPs, trivial and nontrivial surface states, and Fermi arcs have been studied intensively by angle-resolved photoemission spectroscopies (ARPES) and STM/STS. The electronic structures of the top and bottom (001) surfaces are different. In 2016, Bruno et al. reported a surface state in which the Fermi arc emerged out of the bulk electron pocket on the top and bottom surfaces by small-spot laser ARPES [22]. They also stated that the Fermi arc was trivial in topology, and it was not related to the WPs in the bulk band structure, which indicated that it was impossible to observe the Fermi arc solely regarding the WTe_2_ as a WSM in topology. Furthermore, Sánchez-Barriga et al. used ultra-high-resolution ARPES to observe the Fermi arc on the (001) surface at 0.8 K in 2016 [23]. They believed that the Fermi arc was associated with the coupling to the surface resonance dispersing near the boundary of the projected band gap in bulk. They also suggested that it was trivial in topology by comparing the results in experiments and theoretical calculations, but the coupling showed compatibility with the type-II WSM possessing a topological Fermi arc.

In 2016, Wu et al. also investigated the electronic structure of WTe_2_ by ARPES, and confirmed the existence of Fermi arcs that linked the bulk electron and hole pockets on the (001) surface [3]. The WPs were observed at the boundary of the electron and hole pocket, as shown in Figure 4. Also, both trivial and topological areas existed on the same sample surface owing to uneven strain, which proved that the transition from a topological state to a trivial state could be driven by strain. In the same year, Feng et al. also observed and validated the prediction of spin polarization of both the Fermi arc and pockets by spin- and angle-resolved photoemission spectroscopy (SARPES) and theoretical calculations [24]. However, the location of WPs and the Fermi arc were not easy to identify by ARPES, because they stood above the Fermi level. To solve this problem, Lin et al. measured energy-dependent quasiparticle interference patterns by cryogenic STM, and identified that the location of WPs was (*k*_x’_, *k*_y’_, *E*) = (0.23 ± 0.01 Å^−1^, 0.05 ± 0.01 Å^−1^, 50 meV) [25]. The results had good agreement with the theoretical calculations, which proved that WTe_2_ was a type-II WSM. It should be noted that some contrary results about the topological nature of the Fermi arc still exist using ARPES. In 2017, Zhang et al. measured and interpreted the quasiparticle interference pattern of the surface state by STM and first-principles calculations, which confirmed the scattering of surface state [26]. However, they did not validate the existence of WPs in the bulk due to the spin-splitting effect, despite the observation of Fermi arcs.

### 2.4. Chiral Anomaly and Non-Saturable Large MR

The chiral anomaly and non-saturable large MR of WTe_2_ are related to its crystal structure. In Figure 1c, the a-axis (tungsten chain is marked by yellow lines) and b-axis are the two main axes in the crystal structure of WTe_2_ that are mutually perpendicular. A tungsten chain is along the a-axis in the crystal structure [10]. This special Td phase abolishes the inversion symmetry, which is the basis for supporting the existence of Type-II WP, and the interactions of electrons–holes contribute to planar orientation dependence, owing to the inclined band structure. Also, the non-saturable negative MR is observed along the a-axis when the electric field is parallel to the magnetic field, and the current is also perpendicular to the a-axis. Inversely, the positive MR emerges when the unsaturated electric field and the magnetic field are mutually vertical, and the current is parallel to the W-chain [10,27]. This unique phenomenon is associated with the chirality anomalies and plane orientation dependence of WTe_2_. Another reason why the MR is significantly large is that the electrical resistance rises significantly when the direction of the magnetic field is in line with the c-axis, which is absent in the type-I WSM. Furthermore, the flakes should be thicker to suppress the positive MR for the sake of observing this phenomenon. Alternatively, it can also be suppressed by rising pressure, and it can even be eliminated under pressure of 10.5 Pa, which is defined as the critical pressure or transformation pressure [28]. The lattice constant c is decreased during the pressurizing process, but the phase change of the structure is not observed. However, the quantum phase change happens when the pressure is over the critical pressure. According to the research by Krishna and Maitra in 2018, the magnetic field could improve the dimension of hole pockets in the Γ–Z direction, so the topology of the Fermi surface was influenced as well [29]. The MR was suppressed dramatically for the layered WTe_2_ flakes, owing to the decrease of the mobility of electrons and holes [30]. This was also related to the disorder and the amorphous surface oxide layer. Actually, the MR is influenced by doping such as Ga^+^ [31]. The presence of impurities can reduce the concentrations of electrons and holes, thereby changing the band structure and Fermi surface of WTe_2_. Also, the linear MR can be recognized by creating the mobility spectra for weak magnetic fields, and it dominates in a strong field (20 T) [32]. The large MR can be switched on and off by electrostatically doping between a semimetal state and metallic state (only electron) at low temperatures [33].

Research by Ali et al. in 2014 demonstrated that the MR reached 4.527 × 10^5^% at 4.5 K and 1.3 × 10^7^% at 0.53 K and 60 T [34]. As a non-magnetic material, its large MR is different from that of magnetic materials because of the reasonable compensation and equilibrium between the electrons and holes on the Fermi surface below 150 K in the Brillouin zone in the direction of Γ–X. In addition, the balance between the holes and electron carriers also makes the MR sensitive to the pressure, because the pressure tends to influence the lattice structure and balanced state of WTe_2_ [35]. The real carrier concentration can be determined with a large Seebeck coefficient without doping in the heterojunction structure [36]. However, it should be noted that the layered WTe_2_ is not available naturally, and the precursor purifying it is difficult to carry out during the preparation. It also tends to be degraded or oxidized when it is exposed in air, which all increase the difficulty of studying layered dependence properties. By contrast, the MR of WTe_2_ may be improved by up to 30% by the oxidization of the surface in accordance with the research by Li et al. [37].

The sheet-shaped and bulk material should have similar band structures so that WP can exist, and this is attributed to the reverse chirality of coupled WPs. Specifically, a non-zero potential is induced by electrons displacement if the dot product of the magnetic field and the electric field is not zero. The Fermi energy is influenced by the WP that can be controlled by the gate voltage; therefore, the transmission performance is also affected, which expands the applications of WTe_2_ in electronics. In addition, it also has potential as a two-dimensional (2D) contact material due to its relatively low work function.

### 2.5. Metal–Insulator Transitions

**Figure 5:**
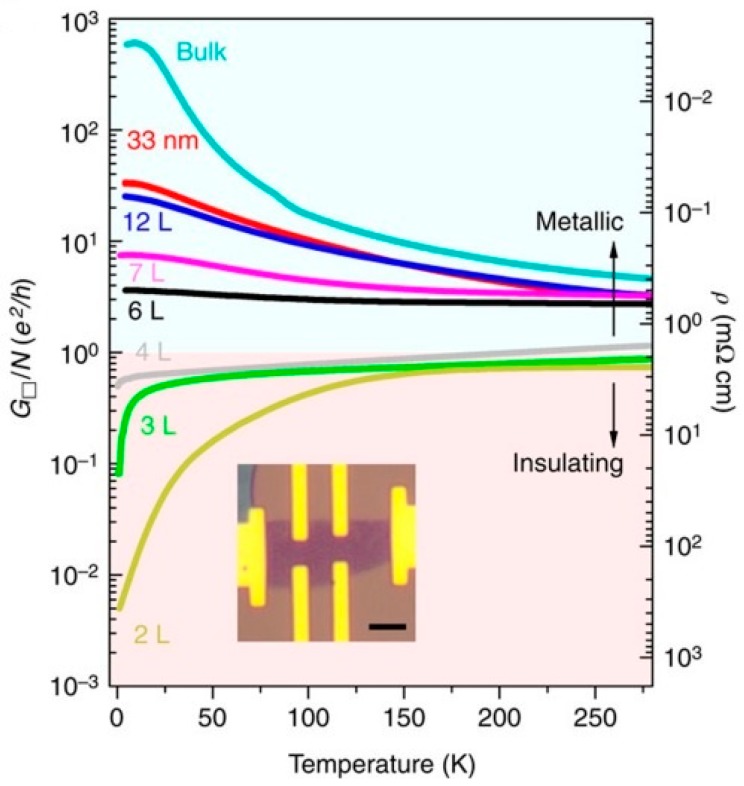
Metal–insulator transition in thin WTe_2_ layer [38].

In 2015, Wang et al. discovered that the conductivity of layered WTe_2_ was associated with its thickness: the conductive resistivity started to transform to the insulated resistivity when the thickness of WTe_2_ was below six layers because of oxidation-induced disorders [38]. The dimension of layered WTe_2_ is not proportional to the number of layers due to the atomic force between layers (i.e., 7.9–8.6 nm for 11 layers) [7]. Also, the degradation or oxidation tends to influence the performance of WTe_2_-based devices, which is especially obvious in the case of fewer layers (i.e., less than four layers). The reason for this is that fewer layers result in a relatively unstable state, so oxidation between layers is severe when the thin WTe_2_ is exposed in the environment during manufacturing, such as during the exfoliation process [38]. As shown in Figure 5, the transport mode of WTe_2_ changes under approximately four layers, and the insulating property dominates when the number of layers is reduced to the limit of the monolayer (each layer owns a conductivity of ~e^2^/h). This is the proof of the metal-insulator transition of conductivity under the temperature dependence [38]. The conductivity is also related to the insulating temperature, especially for the tri and bilayers. It is worth mentioning that the conductivity of the monolayer is significantly low, which is neglected in Figure 5. The above experiment was carried out under low temperature, and the resistivity does not rise considerably under room temperature (i.e., increases five-fold at normal temperature compared to five to six orders of magnitude at T-0.25 K) [38].

The resistivity does not show the layer dependence significantly in the layer range studied. The reason for this may be that the curved W chain interrupts the in-plane 2D symmetry, which is characterized by preferred orientations, so different crystal orientations are performed in the device [7]. The rising interlaminar coupling of lattice distortion causes a moderately anisotropic Fermi surface in the planar layer.

### 2.6. Superconductivity

The superconductivity of WTe_2_ can be induced by increasing the pressure to 10.5 GPa at 2.8 K without changing the phase of the structure, and the large positive MR also disappears with the reversal of the sign of the Hall coefficient at this critical pressure [28]. The increasing pressure increases the number of electron carriers, but decreases the amount of hole carries, and the Fermi surface reconstructs with the change of quantum phase. Actually, the rebuilding of the Fermi surface depends on the anisotropy of lattice, and the a-axis, b-axis, and c-axis are compressed by 0.6%, 3.3% and 6.5% respectively. An interesting thing is that the anisotropy of superconductivity becomes very weak at 98.5 kpa by changing the magnetic field angle [39]. The critical temperature of superconductivity can also be enhanced by in-plane magnetic fields, although it may suppress the superconductivity because of the competition between thermodynamic and magnetic energies [40]. Also, complicated chemical doping can be avoided by controlling the pressure. It should be noted that the transition temperature of superconductivity decreases slightly with increasing pressure (i.e., from 6.5 K-13 GPa to 2.6 K-24 GPa). Furthermore, the pressure variation can also induce the phase change of single crystalline WTe_2_ [41]. The phase changes from orthorhombic T_d_ to monoclinic T′ at 8 GPa because of inverse symmetry, which is in line with the observation of superconductivity.

## 3. Preparation of WTe_2_

Many methods are used to obtain WTe_2_ in different scales and shapes. These methods influence the properties of materials significantly, so they should be investigated to evaluate the availability. Mechanical exfoliation is the most basic method to obtain few-layer to single-layer WTe_2_ in high quality, and it is also applicable for other 2D layered materials such as graphene. However, this method is mainly utilized when large-scale WTe_2_ cannot be obtained, and WTe_2_ tends to degrade upon being exposed in air, so exfoliated flakes are limited to some extent. Other synthesis methods can also be used in real life, which promotes the development of WTe_2_-based devices and research.

### 3.1. Solid-State Reaction

The solid-state reaction can be used to manufacture single crystals of WTe_2_, and the pure powder of W is reacted with the excessive pure powder of Te in the environment of inert gases (i.e., argon) to improve the quality of WTe_2_ [28]. The mixture can be stored in the vacuum quartz container. In addition, the heating temperature is about 1273.15 K, and the mixture should be maintained for 5 h; then, it is cooled down to a lower temperature (973.15 K). In this process, the temperature gradient is very important, and should be controlled properly. The reacted WTe_2_ is finally centrifuged to get the single crystal compound.

### 3.2. Atmospheric Chemical Vapor Reaction

The WTe_2_ samples used in the most experiments are bulk material and flakes fabricated by mechanical exfoliation, because single layer WTe_2_ in good quality can be obtained. However, it is not practical in real applications due to the small-scale limit. In 2017, Zhou et al. firstly fabricated thin-film WTe_2_ by atmospheric chemical vapor reaction directly, and overcame the limitations of low Gibbs free energy (−26.2 kJ·mol^−1^) and the unmatched melting temperature of Te and W [42]. The synthesized polycrystalline WTe_2_ was in high quality, and held low thermal conductivity at 300 K (in plane: 2 W·m^−1^·K^−1^; though plane: 0.8 W·m^−1^·K^−1^), which was significantly lower than single crystalline flakes fabricated by mechanical exfoliation. In 2017, Chen et al. used ambient pressure chemical vapor deposition to synthesize large-scale layered 1T′-WTe_2_ (length of 350 µm) in good quality, and the nanooptical image technique was firstly applied to the WTe_2_ flakes for which the wavelength was obtained (100 nm) [43].

### 3.3. Chemical Vapor Deposition (CVD)

**Figure 6:**
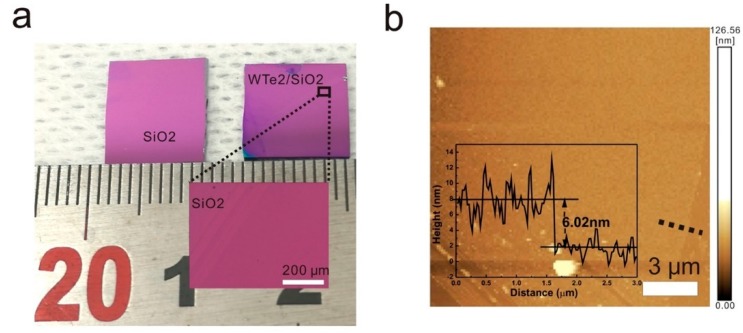
(**a**) The dimensions of the synthesized 1T′-WTe_2_ by chemical vapor deposition (CVD) (SiO_2_/Si substrate); (**b**) atomic force microscopy (AFM) image of the 1T′-WTe_2_ [37].

Chemical vapor deposition (CVD) is a common method that is used to synthesize layered 2D material such as graphene, and small-scale (even atomic scale) WTe_2_ film can also be obtained by this method [27]. In 2018, Li et al. synthesized uniform centimeter-scale (1 × 0.8 cm^2^), large-area WTe_2_ film in a few layers by CVD at low temperature, as shown in Figure 6, and the material was very stable at room temperature for one month despite a conductivity drop [37]. Figure 6b shows the thickness (6.02 nm) of synthesized 1T′-WTe_2_ by means of AFM, and it can be changed by regulating the sources (WCl_6_ powder is selected due to the lower melting point), amount, reacting time, and the reacting distance between WCl_6_ powder and the SiO_2_/Si substrate. The temperature used in this process was relatively low, which was only 500 °C, and the reaction lasted for 20 min at this temperature in the common three-zone system under the protection of N_2_/H_2_. Furthermore, Naylor et al. presented an accessible CVD method to fabricate monolayer 1T’-WTe_2_ flakes, and the surface coverage of this method was around 20%. They also suggested that the degradation of WTe_2_ could be averted by reducing the handling time and introducing few-layer graphene onto the substrate instantly after the reaction to passivate the WTe_2_ [44]. There are two stacking sequences in the bilayer WTe_2_, and low-temperature transport measurements indicate a semimetal–insulator transition, as mentioned [45].

### 3.4. Solution Synthesis

The few-layer Td-WTe_2_ nanostructure (30–50 nm) can be directly synthesized by the solution method at a relatively low temperature (300 °C), and the different growing paths show the multiple stacking motifs [46]. The advantage of this method is that the crystal structure can be adjusted by changing the amount of metal reagent. During the process, the temperature should be controlled properly to improve the purity of material. In 2018, Giri et al. obtained large-area atomically thin WTe_2_ by solution phase preparation [47]. A new Te precursor was also used to change the number of atomic layers (single to few layers).

### 3.5. Other Synthesis Methods

The eutectic metal alloy is a new method for the preparation of single crystalline WTe_2_, which can overcome the high vapor pressure of Te and low binding energy of W-Te [48]. The synthesis parameters of eutectic metal alloys can be studied to improve the quality of the material. The advantage of this method is that the WTe_2_ can be simply transferred to various substrates without degradation. Also, the single crystalline WTe_2_ (band gap is 1.44 eV) could be prepared by direct vapor transport; the orthorhombic structure was observed by x-ray diffraction (XRD) [49].

## 4. Studies of Thermodynamics

The layered WTe_2_ not only has a significantly high current density, it also has ultra-low thermal conductivity, which indicates that it is a potential candidate for applications such as phase change memory electrodes [50]. Many 2D materials such as graphene, h-BN (boron nitride in hexagonal form), and TMDs have attracted the attention of many researchers since 2010. However, only graphene was widely studied, while fewer studies have been carried out for other 2D layered materials. WTe_2_ as a semimetal is an important candidate of TMDs that is very stable in the 1T’ structure, and holds many excellent properties such as low thermal conductivity.

WTe_2_ is characterized by many novel properties, including large unsaturated MR, high carrier mobility, chiral anomaly, superconductivity, etc., as mentioned. By contrast, the large non-saturable MR can only be observed at low temperatures, which limits its application at room temperature. Thermal conductivity is a very important parameter that is related to the thermoelectric efficiency of thermoelectric devices, and studying the heat transfer properties of WTe_2_ is beneficial to improve the design and efficiency of devices. For example, the wasted thermal energy can be collected and reused by thermoelectric materials in ultra-low thermal conductivity applications. Furthermore, the performance of thermoelectric device lies on the merit ZT (figure of merit is defined as *σS*^2^*T*/*κ,* which is related to the Seebeck coefficient *S*, thermal conductivity *κ*, electrical conductivity *σ* and temperature *T*) of material, and dropping the thermal conductivity without changing the electrical conductivity of WTe_2_ is an effective method to increase merit ZT in real life. The ultra-low thermal conductivity can avoid the heat backflow from the high-temperature end to the low-temperature end. It should be noted that WTe_2_ is expected to possess lower thermal conductivity due to heavy atom mass and a low Debye temperature.

### 4.1. Numerical Works

The numerical method is a powerful tool to study the thermodynamic properties of WTe_2_, and most of the studies are based on the theory of the first principle. It is a computational simulation based on DFT, and the commonly used software package is VASP (Vienna ab-initio simulation package) [51]. The Perdew–Burke–Ernzerhof (PBE) of generalized gradient approximation (GGA) is often used as the exchange-correlation function [36,52].

#### 4.1.1. Anisotropic Ultra-Low Thermal Conductivity

WTe_2_ crystallizes in a variant of the CdI_2_ type structure with an octahedral coordination, and the crystal lattice of Td-WTe_2_, called the Td-polytype, contributes to the anisotropy of parameters [53]. According to the first principle study by Gang et al. in 2016, the thermal conductivity peaked at 9.03 W·m^−1^·K^−1^ along the a-axis at 300 K, while it reached its lowest value (0.46 W·m^−1^·K^−1^) along the c-axis [51]. Actually, the cross-plane lattice thermal conductivity is expected to be lower than the calculated value because of its unique layered crystal structure, and it may even be lower than 0.05 W·m^−1^·K^−1^ (the lowest value in solid), which is the planar thermal conductivity of the disordered WSe_2_ (62 nm) thin film [54]. This is a crucial parameter of thermal-insulated material in the thermoelectric devices [51]. In the experiment, the polycrystalline rather than single-crystalline WTe_2_ is usually used as a specimen, and it tends to be influenced by boundary scattering, which is the reason why the anisotropic thermal conductivity is absent in real life. The isotropic thermal conductivity was 0.8 W·m^−1^·K^−1^ in the experiment, and WTe_2_ did not show its intrinsic property [53]. During the simulation process, the structure of WTe_2_ is supposed to be dynamically steady and hold positive values of phonon frequency in the entire Brillouin zone.

In the same year, Ma et al. studied the thermal conductivity of monolayer WTe_2_, and found that the thermal conductivities along the two main lattice directions were 9 W·m^−1^·K^−1^ and 20 W·m^−1^·K^−1^ at 300 K, respectively [8]. This suggested the strong anisotropy of WTe_2_, which emphasized the importance of crystal orientation in the thermal application. The reason for the anisotropy of thermal conductivity is that the linear acoustic phonon branches in the plane, while the out-of-plane secondary acoustic phonon branching shows isotropy. Also, the thermal conductivity of monolayer WTe_2_ was studied by solving the phonon Boltzmann equation (BTE) combined with the first-principles calculation of the acoustic atomic force constant (AFC) in the work by Ma et al. [8]. This phenomenon could not be observed in the bulk material, and it was similar to that of black phosphorous and arsenic, which was related to the planar acoustic phonon mode. This anisotropy is not present in the bulk material, which is associated with the planar acoustic phonon modes.

The anisotropy of intrinsic lattice thermal conductivity was agreed in the research of Sun and Li in 2016, and it stood at 10 W·m^−1^·K^−1^ [52]. However, the thermal expansion could show obvious anisotropy until the temperature was high enough due to the unique properties of the grüneisen parameter and Young’s modulus of the monolayer Td-WTe_2_ [55,56]. Also, the reason why the thermal conductivity of the lattice is anisotropic and extremely low is that the group velocity is small, and the phonon lifespan is relatively short. More phonon modes are activated, and the frequent scattering between acoustic and optical modes reduces the number of high-speed phonons. An important theory is that the thermal conductivity decreases with the dropping characteristic length of the nanostructure (1.5–3.7 μm), which is a potential method to further decrease the thermal conductivity, and the linear thermal expansion coefficient (LTEC) should be also considered in heterostructures. Many properties of semimetal are held by monolayer Td-WTe_2_, which is so large that MR may exist in it as well, which provides the possibility of applications in the magnetic instrument in the nanometer scale [57,58]. The doping can improve the thermoelectric properties of WTe_2_ to some extent. The zero point and vibrational energy will influence the results, so they are included in the analysis, whereas electrons are ignored because of the little contribution to the energy and ultra-low electron density of Td-WTe_2_ (the thickness of a single layer is about 0.7 nm) [59]. The center of the vibration mode changes from 86.9 cm^−1^ in a single layer to 82.9 cm^−1^ and 89.6 cm^−1^ in the bilayer, which means that the vibration is related to the thickness [60]. Actually, the property of anisotropy is reflected in the crystal structure of WTe_2_, which is a distorted orthorhombic structure with octahedral coordination. The atoms in the lattice also slightly divert to the ideal position. For example, a zig-zag W chain is created by the displacement of W atoms along the x-axis, which results in the structural difference in two directions. The distorted structure also affects and combines the acoustic and light modes, which are different from normal TMDs.

#### 4.1.2. Temperature-Dependent Thermal Conductivity

**Figure 7:**
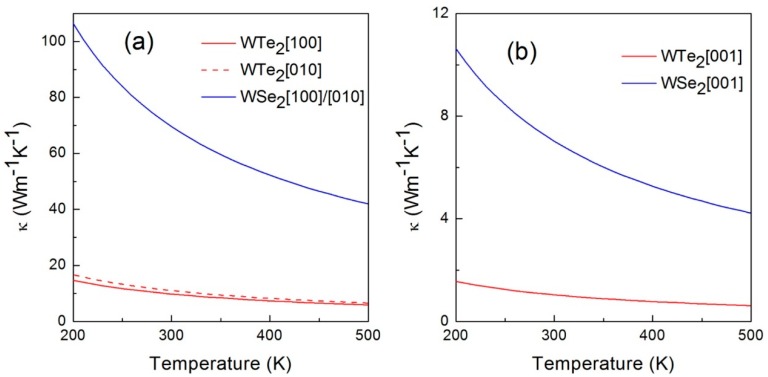
(**a**) The thermal conductivities of WTe_2_ and WSe_2_ along the [100] and [010] directions from 200 K to 500 K; (**b**) The thermal conductivities of WTe_2_ and WSe_2_ along the [001] direction from 200 K to 500 K [51].

The thermal conductivity does not only depend on lattice directions, but also temperature. Figure 7 compares the thermal conductivity of WTe_2_ with that of WSe_2_ in the temperature range of 200 K to 500 K [51]. It is obvious that the thermal conductivities in three directions were all lower than WSe_2_ at the temperature range considered, which suggested the excellent thermoelectrical performance of WTe_2_. Also, the thermal conductivity showed a decreasing trend with increasing temperature because of severe phonon–phonon scattering at high temperature. The difference between the [100]/[010] and [001] directions was significant due to the existence of van der Waals forces in the cross-plane. In addition, the phonon group velocity in the [001] direction was lower than that in the [100] (W–W chain) and [010] directions [52]. The orthogonal symmetry of the crystal structure decides that both [100] and [010] are the major directions of the κ-tensor.

A similar conclusion was interpreted by Ma et al.; the thermal conductivities of monolayer WTe_2_ in the axis of the a and b directions were around 9 W·m^−1^·K^−1^ and 20 W·m^−1^·K^−1^ at 300 K, respectively, and the anisotropy rose from 2.27 (500 K) to 2.44 (100 K) [8]. The averaged square of group velocities (v*_w_*^2^) of single-layered WTe_2_ is about zero, owing to the disappeared secondary branch at the Γ point, which is also different from the constant value of the three-dimensional (3D) material at low frequency. Actually, the significant anisotropy at high temperature is related to the v*_w_*^2^ difference in the a-axis and the b-axis. The v*_w_*^2^ along the a-axis is larger than that along the b-axis at high frequency, but it is reversed at the low frequency. This follows the trend of thermal conductivity, which suggests that low-frequency phonons contribute more to the thermal conductivity. Therefore, the increasing anisotropy is observed at decreasing temperature.

#### 4.1.3. Temperature-Dependent Thermal Expansion and Mechanical Properties

In 2016, Sun and Li found that the mechanical properties, such as the modulus of elasticity *E* of WTe_2_, were associated with the temperature change based on calculations of free energy [52]. The in-plane anisotropic mechanical properties were influenced by the phonon excitation. For example, Poisson’s ratio in the [100] and [010] directions dropped with rising temperature, which demonstrated that the opposite effect was employed to the in-plane stiffness and weaker strain correlation. Also, the levels of in-plane anisotropy were different for monolayer Td-WTe_2_ from 0 K to 600 K, in that Young’s modulus increased significantly along the x-axis but almost remained the same along the y-axis (*E*_x_ is smaller than *E*_y_ in the entire temperature range). This is unique to the 2D materials such as graphene; *E* decreases as temperature rises because of the reduced curvature of the potential energy surface induced by thermal expansion. The phonon excitation is the possible reason for the strengthened *E*.

In terms of thermal expansion, it is associated with the non-harmonic interactions, similarly to the thermal conductivity, while the characteristic of anisotropy of thermal expansion is slightly different with that of thermal conductivity. According to the calculated LTEC, the value was positive along the [100] and [010] directions from about 0 K to 600 K (only a very small negative value is observed at 0 K), which was in line with the other TMDs, but significantly different from some 2D materials, including h-BN and graphene [52]. In addition, the difference between thermal expansions in two directions is not considerable until the temperature increased above 150 K, but the increase rate is higher at low temperature due to active phonons and the slight extension of the frequency. Furthermore, the thermal expansion of a single layer WTe_2_ is influenced by in-plane stiffness and charge transfer. In 2015, Wang et al. found that negative thermal expansion was associated with bending modes in monolayer WTe_2_ rather than bulk material at low temperature according to first-principles calculations, and the positive value was mainly related to in-plane stiffness [61]. Overall, the unique LTEC shows that single-layered WTe_2_ can facilitate the design of heterojunction devices.

#### 4.1.4. Scale-Dependent Thermal Conductivity

**Figure 8:**
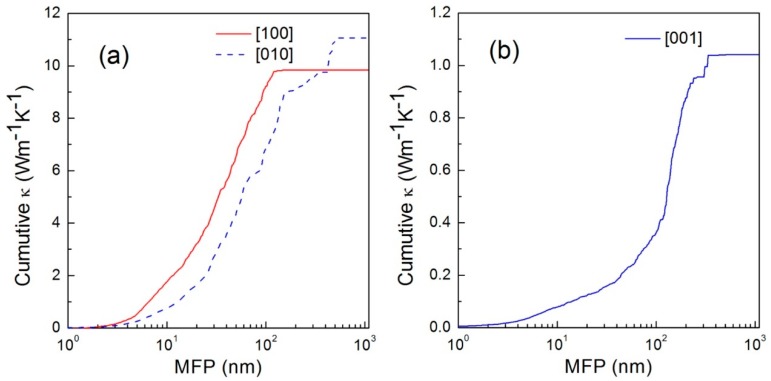
Cumulative thermal conductivity—phonon mean-free path (MFP) graph in WTe_2_ in the x, y (**a**) and z (**b**) directions, respectively [51].

The thermal conductivity of WTe_2_ also depends on the dimensions, and the cumulative value can be used to evaluate the scale dependence. Figure 8 shows the graph of cumulative thermal conductivities versus mean-free paths (MFP) in three main directions [51]. Obviously, a larger scale contributed to higher thermal conductivity in three directions, but the relation was non-linear, showing a platform with various threshold values when the MFP was over 100 nm. An interesting phenomenon was that the cumulative thermal conductivities along the a-axis and b-axis showed stronger dependence than the c-axis direction before 100 nm, due to the considerable suppression of low-frequency phonons along the b-axis. This indicates that reducing the scale of WTe_2_ will further decrease the thermal conductivity. However, it can be reduced infinitely, because the conductivity will also drop with small dimensions, which limits the performance of the nanodevices. It should be noted that doping such as Mo is a reasonable method to improve the conductivity and thermoelectric efficiency.

The scale dependence of thermal conductivity is effective even up to 10 μm (sample size along the a-axis or b-axis) owing to low-frequency phonons, and the anisotropy of small-scale material is not as significant as that of large-scale material because of the increasing boundary scattering in the nanostructure and anisotropic phonons at low frequency [8]. This is similar to the results of the research by Gang et al. The boundary scattering effect can suppress the thermal conductivity, especially in nanostructures and at low temperature.

#### 4.1.5. Specific Heat and Debye Temperature

The heat capacity of WTe_2_ can be measured by adiabatic calorimetry in the temperature range of 5.5 K to 329 K, in which phase transition is absent. The molar heat capacity showed the abnormal increasing trend between 92 K to 175 K, and the electron value, as well as the Debye temperature, stood at (5.99 ± 1.83) mJ·K^−1^·mol^−1^ and 133.8 K, respectively [62]. Also, standard molar thermodynamic functions were observed in an interesting range of temperatures. The Debye temperature that is related to many physical properties such as the melting temperature does not change theoretically for all crystal at high or low temperatures with regard to the Debye model, in spite of the certain error in real life. The minimum value is generally determined from two extremes at a lower temperature. However, the specific heat and Debye temperature of WTe_2_ are temperature-dependent [51]. They both increase and approach the threshold value with the rising temperature (range of 0–500 K) because of low-temperature T^3^ law behavior (Dulong–Petit limit). The simulated value is generally lower than the experimental value, owing to the thermal expansion induced by the non-harmonic effect. The increase rate of lattice heat capacity drops at high temperature.

### 4.2. Experimental Works

**Figure 9:**
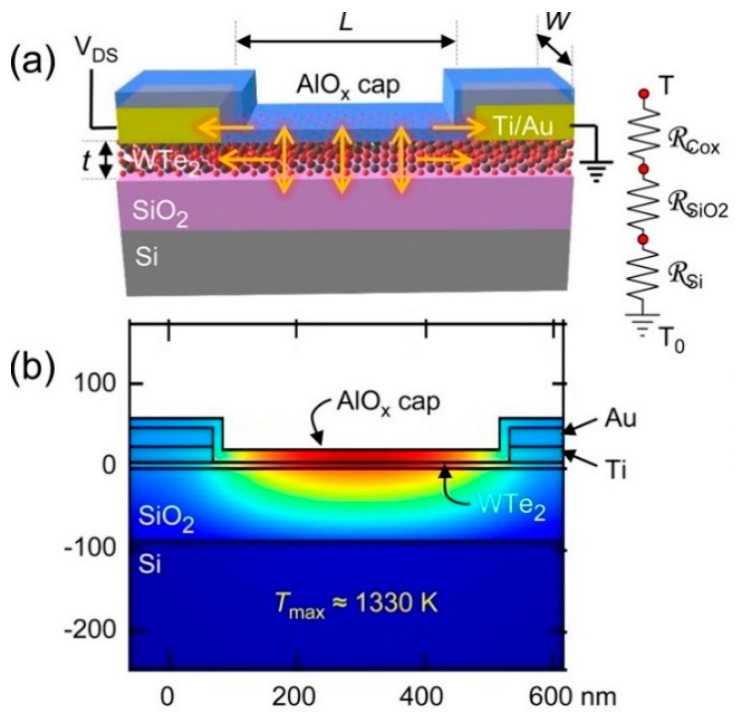
(**a**) Schematic of WTe_2_ device in the experiment; (**b**) The simulation of thermal property of WTe_2_ before breakdown [7].

In addition to simulation, the experiment is also a common method to study the thermodynamics of WTe_2_. In 2016, Xu et al. studied the thermal limit of 2D semimetal few-layer WTe_2_ devices onto Si substrates in vacuum condition [50]. The device was covered by AlO_x_ cladding, which was acting as a heat sink to support high current density (over 40 MA/cm^2^) and avoid the oxidation. The thermal conductivity and thermal interface resistance were estimated using electrothermal theory, and the maximum temperature range (melting temperature was 1300 K) was also considered during experiment and simulation. Finally, the estimated thermal conductivity was approximately 3 W·m^−1^·K^−1^ to 10 W·m^−1^·K^−1^. Ongoing research was carried out by Mleczko et al. in the same year, in which the current density and thermal conductivity of atomic-scale layered WTe_2_ films were studied experimentally [7]. The sample with three to 20 layers was isolated from the air during preparation to avoid degradation. Figure 9a,b show the schematic of the WTe_2_ device used in the experiment and simulation, respectively [7]. The perpendicular thermal resistance model represented the corresponding heat flow, and the simulation was used to validate the analytical results. The flow path (arrows) of heat was also shown in Figure 9a, and the substrate consisted of SiO_2_ and Si at the ambient temperature. The lateral heat dissipation through the AlO_x_ capping should be considered before the experiment. A method that could avoid the influence of contact resistances and perform the intrinsic property in the low-field measurement was using small-scaled specimen at low temperature (80–300 K). This reduced the difficulty of investigating a thin but stable WTe_2_-based device. They also found that few-layered WTe_2_ could support higher current (50 MA/cm^2^) density than most interconnect metals, and the thermal conductivity in the [100] direction was as low as 3 W·m^−1^·K^−1^ through contrasting with the self-heated model. The thermal conductivity was determined from the difference of the effective value of the device and the thermal conductivity of the AlO_x_ cap.

The electrons contribute 10–30% to the measured thermal conductivity in accordance with the Wiedemann–Franz law. The asymmetry of the structure also shows the significant influence of the thermal conductivity, but the anisotropy cannot be observed directly. Assistant techniques such as orientation mapping and assisted measurement techniques are needed to determine the parameters in different directions. Furthermore, the characteristics of high current density and low thermal conductivity make WTe_2_ become a potential candidate of applications of thermoelectric materials, while its thermal power (Seebeck coefficient) is relatively small, which should be studied and improved from the electronic structure in the future. Also, it can be used as an electrode in the phase change memory, so that the programming energy per bit can be reduced, and it can act as a 2D contact in layered transistors.

The WTe_2_ used in the experiment is generally small-scale, because large-scale material tends to have more defects and is difficult to obtain. Zhou et al. prepared a large-scale polycrystalline WTe_2_ thin film directly with low thermal conductivity by atmospheric chemical vapor reaction in 2017 [42]. The W metal film was reacted with Te vapor under the catalyzing of the H_2_Te in low bonding energy, which provided a revelation of preparation of single crystal deuteride nanoplatelets. The measured thermal conductivities along the a-axis and b-axis were all less than 2 W·m^−1^·K^−1^ at 300 K, which was considerably lower than that (15 ± 3 W·m^−1^·K^−1^) of single crystal WTe^2^ sheet fabricated by mechanical exfoliation. The in-plane anisotropy was not observed in single crystal WTe_2_, and it might be impossible to find in the experiment. In addition, the thermal conductivity in the direction of the c-axis stood at 0.8 W·m^−1^·K^−1^ at 300 K due to the suppression of small grain size and distorted crystal structure. Specifically, the grain boundary and distortion of the polycrystalline material can suppress the effects of phonons and electron transport, so the electrical and thermal conductivities of film are lower than the bulk material. The influence of electrons on thermal conductivity is anticipated to be very small (only around 1.0 W·m^−1^·K^−1^). The atomic mass of WTe_2_ is heavy, and its symmetry is low in the plane, so its thermal conductivity is expected to be lower than other TMDs such as WSe_2_ [51].

## 5. Summary and Analysis

WTe_2_ as a type-II semimetal is a typical candidate of TMDs and has many excellent properties, including novel Weyl fermions, Fermi arcs, surface state, chiral anomaly, non-saturable large MR, pressure-induced superconductivity, high current density, ultra-low thermal conductivity, metal–insulator transitions, etc. It is a new generation of topological insulators, and also has a unique band structure both on the surface and in the bulk, and its phase is between an ordinary and topological phase.

In addition, it has a distorted 1T (Td) structure, which is different from other TMDs that have 2H or 1T structures at room temperature, and the asymmetric crystal structure indicates its anisotropic physical properties. The wide interlayer distance means that the properties of WTe_2_ tend to be easily influenced by doping such as Ga^+^. Graphene has been widely studied in recent years, but WTe_2_ has not been sufficiently researched due to its late discovery. Most reports are about the characteristics of chiral anomaly and non-saturable large MR, but WTe_2_ is also a promising non-magnetic thermoelectric semimetal that holds extremely low thermal conductivity. WTe_2_ can be used in applications of low dissipation electronics, semiconductors, spintronics, optoelectronics, thermodynamics, catalysis, etc.

The Weyl fermions in the WTe_2_ can be directly observed by photoelectron spectroscopy in the experiment, which breaks the limit of low-energy excitation. As a type-II WSM, the paired WPs locate at the boundaries between electron and hole pockets, which is unique to type-I WSM. The surface state can be observed by ARPES and studied by quasiparticle interference pattern. Although there are controversies about the topological nature of the Fermi arc surface state, WTe_2_ has still attracted intensive attention in recent years. Also, the special crystal structure contributes to the chiral anomaly and non-saturable larger MR. The equilibrium between electrons and holes results in large MR, and negative as well as positive values are observed when the applied electric field is parallel and perpendicular to the magnetic field, respectively. In addition, increasing the thickness or pressure of WTe_2_ flakes can suppress the positive MR. The MR is as high as 4.527 × 10^5^% at 4.5 K, and 1.3 × 10^7^% at 0.53 K and 60 T [34]. Furthermore, the change of thickness (below six layers) can cause metal–insulator resistivity transitions, and the conductivity is affected by temperature. Another promising property is pressure-induced superconductivity that occurs under pressure of 10.5 GPa at 2.8 K, which corresponds to the absence of positive MR.

The preparation methods will influence the properties of WTe_2_ significantly. It is relatively difficult to prepare high-quality small-scale WTe_2_ because of the high equilibrium vapor pressure of Te and low binding energy of W-Te [48]. The layered WTe_2_ does not exist in nature, so the materials used in the experiment are generally prepared by mechanical exfoliation, CVD, and solid-state reaction to improve the purity. However, it is relatively difficult to purify the precursor during preparation, and WTe_2_ is easy to be oxidized or degraded once it is exposed in the air, so it is generally isolated from the air or protected by the inert gas, which improves the difficulty and resources needed in the experiment to some extent. It should be noted that spectroscopic analysis and the chemical bonding of the vibration mode may prevent few-layer WTe_2_ from the influence of degradation [7]. Furthermore, mechanical exfoliation is the most basic method to manufacture small-scale monolayer or few-layer WTe_2_ in high quality, but the extra process should be carried out to prevent degradation. It is not suitable for large-scale fabrication, so it is mainly used in experiments. CVD is the most promising method for the synthesis of large-scale, large-area layered WTe_2_, and few-layer graphene can reduce the oxidation during manufacturing. In addition, atmospheric chemical vapor reaction is a direct method to synthesize polycrystalline WTe_2_ with low thermal conductivity in good quality, and solution synthesis is expected to be applicable in doping engineering. Some new methods, such as eutectic metal alloys and direct vapor transport, promote the development of WTe_2_ as well. Overall, although the large-scale few-layer WTe_2_ can be obtained in high quality, mass production still cannot be achieved, which limits its application in real life, and CVD is a potential method for manufacturing in industry.

WTe_2_ is a promising non-magnetic thermoelectric semimetal, and its thermodynamic properties are summarized in this work. The common research methodologies of WTe_2_ are numerical simulation, such as first-principle calculations and experiments. The results of the first principle are associated with the models used in the simulation. The accuracy will be improved if the interlaminar force (van der Waals) is included. Basically, the empirical correction scheme, such as Grimme, can improve the accuracy of results, which is verified by the calculations of lattice parameters, phonon dispersion, and density of state compared with experimental values [51]. Also, extra considerations such as vacuum along the z-axis (more than 23 Å) should be considered in order to minimize the interactions between periodical images in the case of monolayer Td-WTe_2_ [52]. Layer separation thickness (7.009 Å) of bulk WTe_2_ is employed to remedy the deficiency of the volume of the unit cell in the 2D system [8]. A cut-off value of 0.5 nm should be considered for the calculation of non-equilibrium atomic force constants because of the special distorted orthorhombic structure, and the results of thermal conductivity must be converged in this value.

The thermodynamic properties of WTe_2_ exhibit strong anisotropy due to its unique distorted crystal structure. Table 1 shows the thermal conductivities of WTe_2_ along the [100], [010], and [001] directions. The thermal conductivity can be calculated from the ratio of heat flux and gradient of temperature, which is a very important parameter of thermoelectric devices. Its value along the y-axis is approximately twice as high as that along the W–W (a-axis) chain, and it shows a decreasing trend with increasing temperature along both axes due to significant phonon scattering at higher temperatures. Table 1 shows that the thermal conductivities in the [100], [010], and [001] directions are 9.03 W·m^−1^·K^−1^, 7.69 W·m^−1^·K^−1^, and 0.46 W·m^−1^·K^−1^ at 300 K, respectively, in the research by Gang et al. [51]. The mean value (1.35 W·m^−1^·K^−1^) was also evaluated by the Mathiessen rule, which was higher than the experimental value standing at 0.8 W·m^−1^·K^−1^ owing to microdefects and boundary scattering in polycrystalline specimens [53]. The lattice thermal conductivity is related to the phonons behavior according to the Boltzmann transport theory. Also, the thermal conductivity along the c-axis is the lowest among the three main directions owing to van der Waals forces, and it is expected to be even lower than that of WSe_2_ (the lowest value in solid) because of the heavier atom mass and lower Debye temperature. Furthermore, the thermal conductivity determined from first-principle calculations and experiments is slightly different due to the defects and boundary scattering. It is also difficult to observe in-plane anisotropy in the experiment, because the polycrystalline material is usually used, so that the intrinsic property is suppressed. The reason why the thermal conductivity is extremely low is due to the small group velocity and short phonon lifespan. Moreover, compared with other 2D materials such as h-BN and graphene, the thermal conductivity is one to four orders of magnitude lower, which shows the excellent thermoelectric properties of WTe_2_.

The thermal conductivity not only depends on lattice orientations, but also on temperature and scale. It decreases with increasing temperature due to significant phonon interactions, but severe anisotropy is shown at high temperature, because of the v*_w_*^2^ difference in three directions. Also, larger dimensions induce higher thermal conductivity, and the threshold value is exhibited in three directions around 100 nm. The thermal conductivities along the a-axis and the b-axis are significantly influenced by scale due to the suppression of low-frequency phonons. This indicates that the thermal conductivity can be further decreased by reducing the scale to nanometers, but the scale limitation of conductivity should be considered as well to maintain the performance of WTe_2_-based thermoelectrical devices. In addition, doping such as Mo is expected to solve this problem. Another consideration is that the dimensions cannot be reduced infinitely, and the significant boundary scattering will influence the thermal conductivity, especially in nanostructures or at low temperature.

Other thermodynamic properties also show temperature dependence including Poisson’s ratio, Young’s modulus, and thermal expansion influenced by phonon excitation Poisson’s ratio decreases at a higher temperature. By contrast, the modulus of elasticity rises at high temperature and shows stronger temperature dependence in the [100] direction than the [010] direction, which was different from graphene. As for thermal expansion, it is positive and increases from 0 K to 600 K because of non-harmonic interactions, although it shows a very small negative value at 0 K. In addition, the thermal expansions are different in two directions after 150 K, and this property is influenced by in-plane stiffness and charge transfer. Overall, the unique thermal expansion suggests that single-layered WTe_2_ can be used in the design of heterojunction devices.

WTe_2_ owns high current density and low thermal conductivity, which improves its availability in the thermoelectric devices. However, as a typical semimetal, its low thermal power (Seebeck coefficient) should be improved in the future. It can also be used in the applications of phase change memory; the WTe_2_-based electrode can reduce the programming energy per bit. In addition, it can act as a 2D contact of layered transistors in the magnetic memory, sensors, and spintronics.

## 6. Conclusions

WTe_2_ is a promising type-II WSM and new generation of topological insulator holding many excellent properties including chiral anomaly, non-saturable large MR, pressure-induced superconductivity, ultra-low thermal conductivity, etc. Unlike some 2D materials such as graphene, it is not sufficiently studied, especially for thermodynamics, due to its late discovery. Its thermodynamic properties such as thermal conductivity depend on the temperature, scale, and lattice orientations, and WTe_2_ may hold the lowest thermal conductivity in the solid owing to the heavy atom mass and low Debye temperature. Also, the massive manufacturing of large-area few-layer WTe_2_ in good quality is not achieved at present, which limits its development and application. In conclusion, WTe_2_ is a promising material that can be used in applications of low-dissipation electronics, semiconductors, spintronics, optoelectronics, thermodynamics, catalysis, etc.

## Figures and Tables

**Table 1:** The thermal conductivities κ of WTe_2_ along different directions.

Material	[100] (W·m^−1^·K^−1^)	[010] (W·m^−1^·K^−1^)	[001] (W·m^−1^·K^−1^)	T (K)	Methods	Ref.
Monolayer WTe_2_	9	20	-	300	First-principle	[8]
Td-WTe_2_	9.03	7.69	0.46	300	First-principle	[51]
Monolayer Td-WTe_2_	10	20	-	300	First-principle	[52]
Atomically thin WTe_2_	2.5–3.5 to 9–11 in the [100] direction			80–300	Experiment	[7]
4–20 layers Td-WTe_2_ flakes	3–10			80–300	Experiment and theory	[50]
Few-layer Td-WTe_2_	0.96–1.06 (total κ), 0.8 (mean κ)			300–623	Experiment	[53]
Polycrystalline WTe_2_ film	Less than 2		0.8	300	Experiment	[42]
Single-crystal WTe_2_ flake	15 ± 3					
WSe_2_ (62 nm thick)	0.048 ([001], the lowest value in solid)			300	Experiment and simulation	[54]
Monolayer WSe_2_	50	50	6	300	First-principle and theory	[63]
Single-crystal WSe_2_ platelets	9.7	9.7	2.09	300	Experiment	[64]
ReS_2_ flakes (60–450 nm)	70 ± 18	50 ± 13	0.55 ± 0.07	300	Experiment	[65]
MoS_2_ transistors	14 ± 4			300	Experiment	[66]
Single-layer h-BN	600			300	Numerical solution	[67]
Single-layer graphene	About (4840 ± 440) to (5300 ± 480)			300	Non-contact measurement	[68]
